# Response of negative ion beamlet width and axis deflection to RF field in beam extraction region

**DOI:** 10.1038/s41598-024-81334-w

**Published:** 2025-01-16

**Authors:** Kenichi Nagaoka, Haruhisa Nakano, Taiga Hamajima, Ryoya Nakamoto, Katsuyoshi Tsumori, Masaki Osakabe, Masashi Kisaki, Kenji Miyamoto, Kazunori Takahashi, Ursel Fantz

**Affiliations:** 1 National Institute for Fusion Science, Toki, 509-5292 Japan; 2 Nagoya University, Nagoya, 464-8602 Japan; 3 Present Address: Denso cooperation, Kariya, 448-8661 Japan; 4 Present Address: IHI cooperation, Yokohama, 235-8501 Japan; 5 Nagaoka University of Technology, Nagaoka, 940-2188 Japan; 6 The Institute for Advanced Science, SOKENDAI, Toki, 509-5292 Japan; 7 Present Address: National Institute for Quantum Science and Technology, Naka, 311-0193 Japan; 8 Naruto University for Education, Naruto, 772-8502 Japan; 9 Department of Electrical Engineering, Tohoku University, Sendai, 980-8579 Japan; 10 Max-Planck Institute for Plasma Physics, Garching, 85748 Germany

**Keywords:** Sheath with negative ion, RF negative ion source, Perveance, Physics, Applied physics, Plasma physics

## Abstract

Beam-divergence characteristics of single negative ion beamlet have been experimentally investigated with a superimposition of a controlled perturbation of a radio frequency wave (RF) field in a filament-arc discharge negative ion source. Oscillations of a negative-ion beamlet width and axis responding to the RF perturbation were observed, which may be a cause of the larger beam divergence angle of the RF negative ion source for ITER. It is pointed out that the oscillation of the beamlet width depends on the perveance and on an RF frequency such that the oscillation is suppressed at perveance-matched conditions and at low RF frequency.

Negative hydrogen ions have a large cross-section for charge exchange in a high-energy regime (typically more than 50keV for hydrogen and 100 keV for deuterium), and negative ion beams have been utilized in particle physics experiments, magnetically confined fusion experiments, small-size accelerators for medical applications, etc. In order to expand their applications widely and to make breakthroughs in scientific research projects, further improvements in negative ion source performances such as long pulse operation capability, higher beam current density, better beam optics, etc are going on.

Recently, it has been recognized that beam divergence of the radio frequency wave (RF) negative ion source for ITER^1–8^ is larger than that of filament-arc negative ion sources, while it can almost satisfy the requirement for long pulse and high beam current density operation. The beam divergence of the RF negative ion source is 9-12 mrad at an acceleration voltage of 50 kV^9,10^, and that of the filament-arc source is 5 mrad at almost the same acceleration voltage. While the beam divergence may improve at 1 MeV acceleration^11^, improvement of beam divergence is an important and urgent issue for the development of the RF negative ion source for ITER from viewpoints of avoidance of damage in beamline components and the grid system in the accelerator^12^.

Behaviors of the negative ion sheath near the beam extraction region play an important role in negative ion beam optics because the negative ion sheath boundary, the so-called ”plasma meniscus,” is an electrostatic lens at the first stage of negative ion beam acceleration. However, a physics model of negative ion sheath formation has not been established yet. Therefore, fundamental beam optical characteristics have been investigated with experiments and numerical modellings so far^13–18^.

An important finding is the oscillating behavior of the negative ion beam in the accelerator at the Japan Proton Accelerator Research Complex (J-PARC), which is an RF-negative hydrogen source with an RF frequency of 2 MHz. The beam width oscillated at 2 MHz and a beam current oscillation at 4 MHz was also observed^19,20^. The oscillation at 2 MHz is understood as a direct response of the meniscus to the RF field, and the oscillation at 4 MHz is considered to be related to plasma production. Because inductive electric field intensity reaches the maximum value twice in one RF cycle, then the density fluctuation in the ion source may have a second harmonic component. The RF antenna of the J-PARC ion source is located inside the vacuum vessel and in the vicinity of the plasma grid aperture, thus, both effects by the RF field (a direct RF field effect and a plasma production effect) were observed. The RF antenna configuration for ITER is different from the J-PARC source, and the RF antenna is located outside the vacuum vessel. Plasma diffusion in a filter magnetic field, which has a longer time scale than RF frequency, may dominate the plasma dynamics near the PG, thus, the direct RF field is considered to be more important than the density fluctuation in the RF source for ITER.

In order to clarify the direct RF effect on beamlet divergence and investigate the detailed characteristics, beamlet dynamics were investigated when a controlled perturbation of the RF field was applied to the filament-arc negative ion source. From the viewpoint of difference from the J-PARC source, the plasma production effect was minimized in this experiment due to independent control between plasma production and RF perturbation.

The present study experimentally shows that the beam oscillation and divergence are induced by the RF electric field near the PG aperture, while it is demonstrated that the RF-induced divergence can be minimized by the proper operation of the negative ion source at the perveance matching condition. The present discovery suggests that minimizing the influence of the RF electric field and the elaborate design and operation of the beam extraction with the perveance matching condition contributes to satisfying the requirement for ITER neutral beam injection (NBI) and expands the application of the negative ion beams.

## Results

### RF perturbation in negative ion source

The experiment was carried out with a negative ion beam test stand at the National Institute for Fusion Science (NIFS-NBTS), and a research development negative ion source (NIFS-RNIS) was utilized, which is a one-third scaled negative ion source for the Large Helical Device (LHD).

**Fig. 1:**
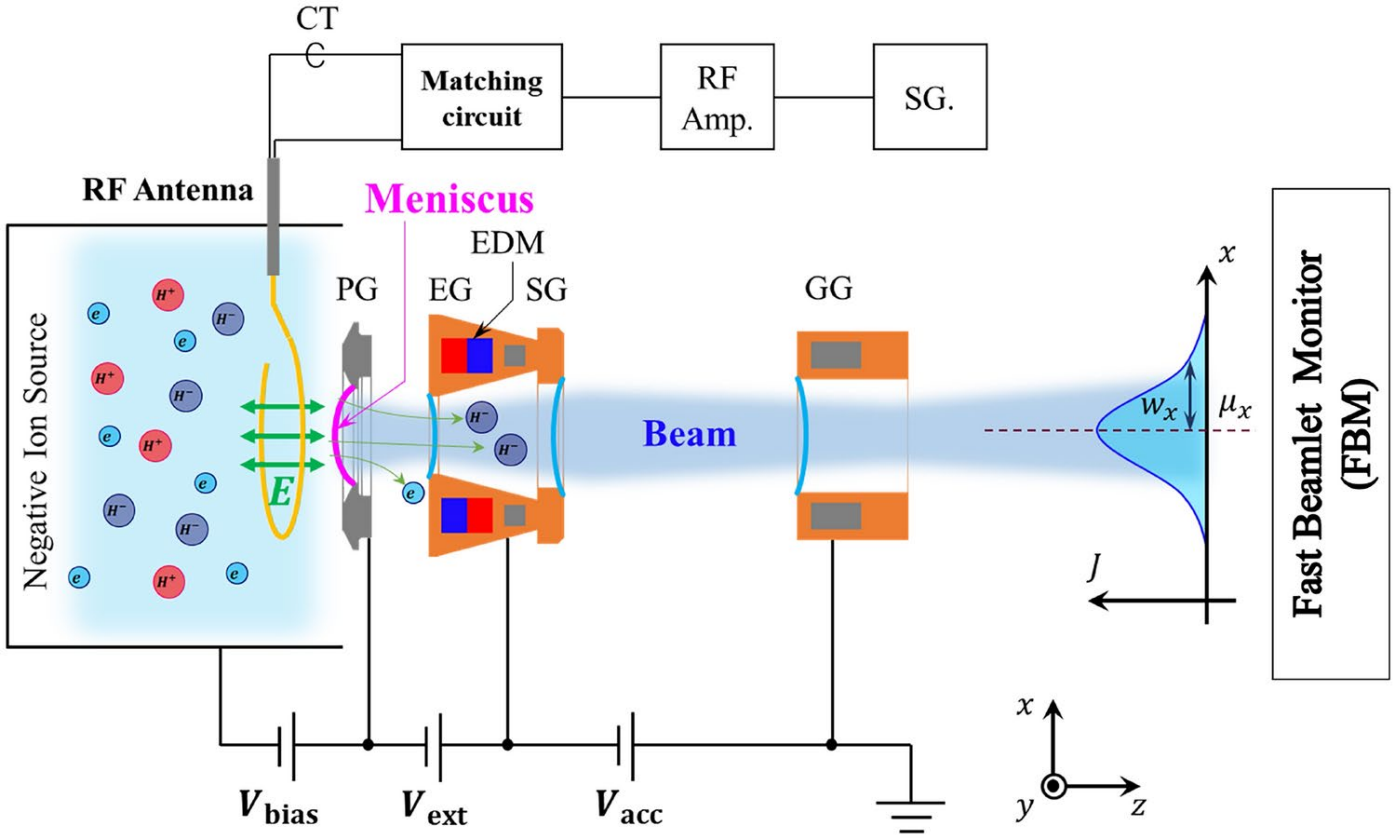
A schematic of the experiment.

A schematic of this experiment is shown in Fig. 1. A Rogowski coil-type RF antenna was installed into NIFS-RNIS and the distance from the PG was around 5 mm. The RF electric field applied by the antenna in this experiment was perpendicular to the PG, which is considered to be the same configuration as the RF negative ion source configuration. The RF signal produced by a signal generator was amplified by a solid-state RF amplifier with a factor of $10^{6}$ (a gain of 60 dB) and the maximum power was 1 kW, which was typically $2\%$ of the arc discharge power ($\sim 50$ kW), resulting in negligible perturbation of the plasma density in this experiment. The RF power to the antenna was monitored by a current transformer (CT) with high-time resolution inserted between the antenna and the impedance-matching circuit. A PG mask was utilized to make a single isolated beamlet. The beamlet profile was measured with a fast beamlet monitor (FBM) with a time resolution of 25 MHz. The FBM was installed at 0.91 m downstream from the grounded grid (GG), which is the end grid of the negative ion accelerator.

**Fig. 2:**
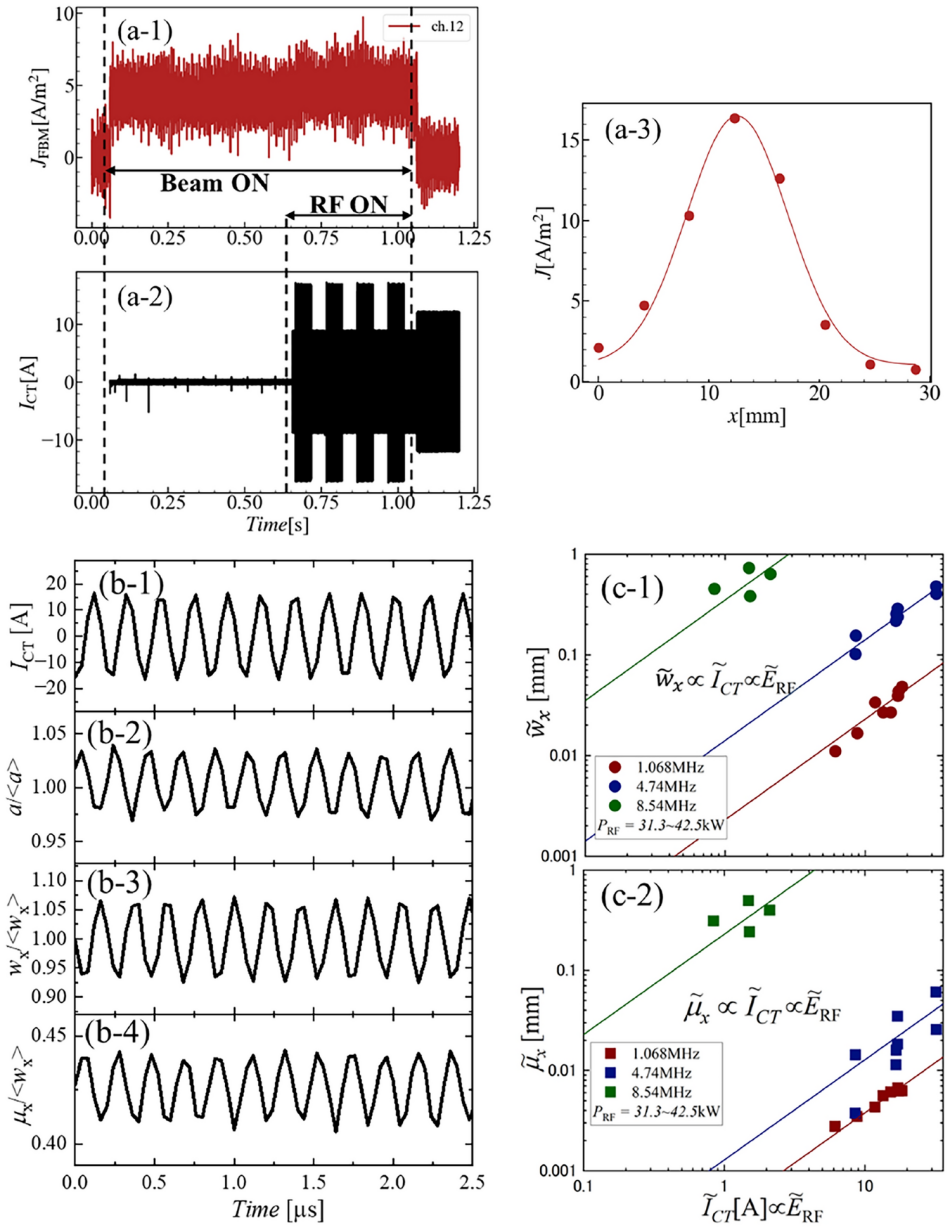
A typical waveform of (**a-1**) the beam current density measured with 12ch of FBM ($J_{\textrm{FBM}}$) and (**a-2**) current of RF antenna measured with current transformer ($I_{\textrm{CT}}$). The power of perturbation RF is modulated. (**a-3**) Typical horizontal profile of beamlet measured by the fast beamlet monitor (FBM). Red line shows a result of Gaussian fitting. Time evolutions of (**b-1**) RF antenna current measured by the CT, (**b-2**) the peak value, (**b-3**) e-folding half width and (**b-4**) the axis position of the Gaussian fitting to experimentally observed negative ion beam current density profile. The bracket $<>$ means the average value in time. RF current dependence of the oscillation amplitude of (**c-1**) the beamlet width and (**c-2**) beamlet axis position. The horizontal axis is the RF current amplitude of RF antenna (${\tilde{I}}_{\textrm{CT}}$) measured by the current transformer (CT), which could be proportional to the RF electric field (${\tilde{E}}_{\textrm{RF}}$). Lines show the linear relation, that is, $\tilde{w}_{\textrm{x}} \propto \tilde{I}_{\textrm{CT}}$ or $\tilde{\mu }_{\textrm{x}} \propto \tilde{I}_{\textrm{CT}}$.

After the commissioning operation with caesium seeding, a negative ion beam acceleration with the perturbation RF field was carried out. The negative ion beam duration was 1 sec, and the perturbation RF was applied during the last one-third period of the beam. The RF power was modulated in time to investigate the RF power dependence. A typical waveform of the beam and RF perturbation is shown in Fig. 2a.

**Fig. 3:**
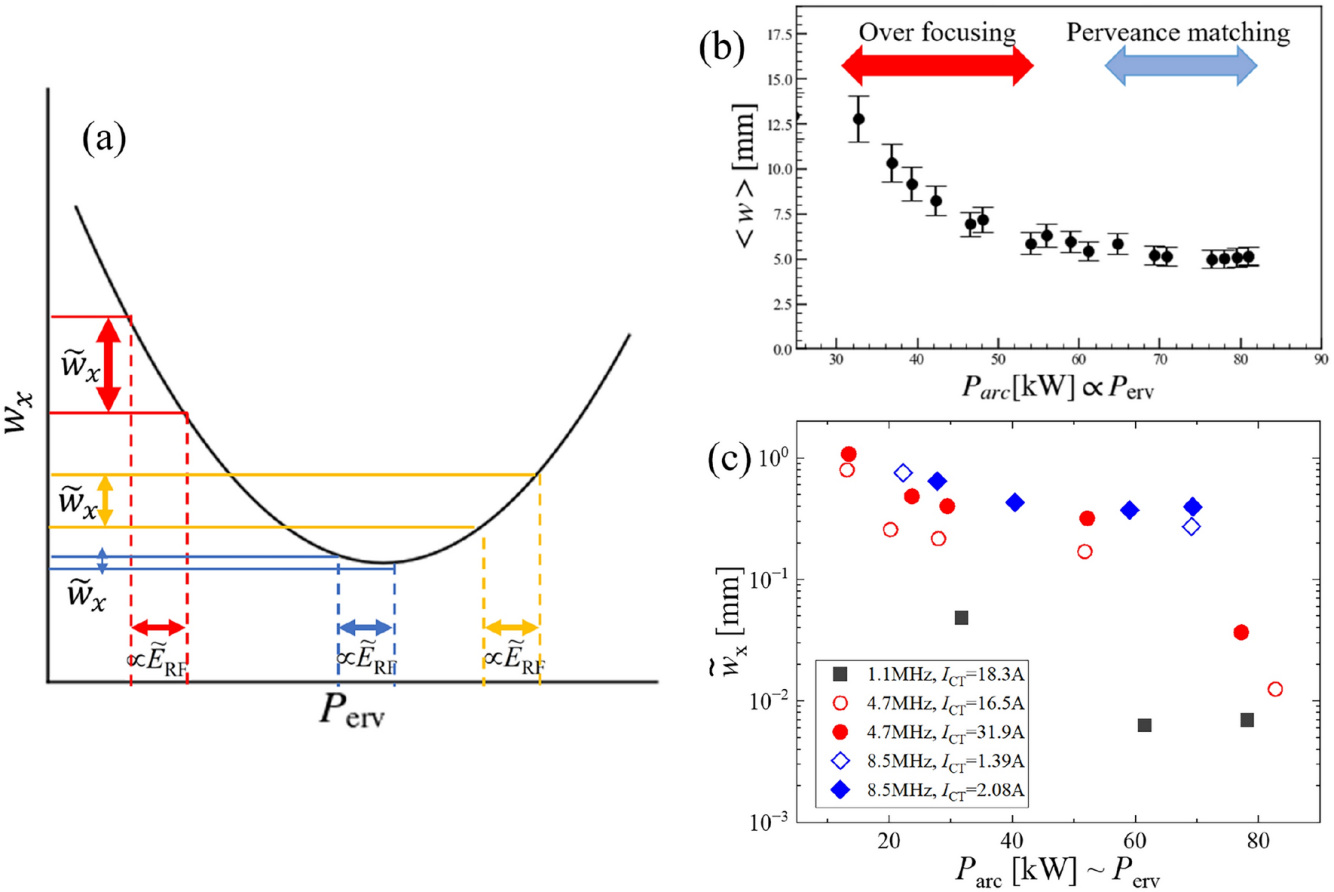
(**a**) A conceptual drawing of the dependence on the gradient of the perveance curve shown in Eq. 2. The relation between beamlet width oscillation (${\tilde{w}}_{\textrm{x}}$) and the perturbation RF electric field (${\tilde{E}}_{\textrm{RF}}$) are also shown. (**b**) Arc-power dependence of the beamlet width without RF perturbation. The beam current is proportional to the arc power in this operational regime in the present experiment. Thus, it can be regarded as a perveance dependence. (**c**) Arc-power dependence of the oscillation amplitude of the beamlet width.

**Fig. 4:**
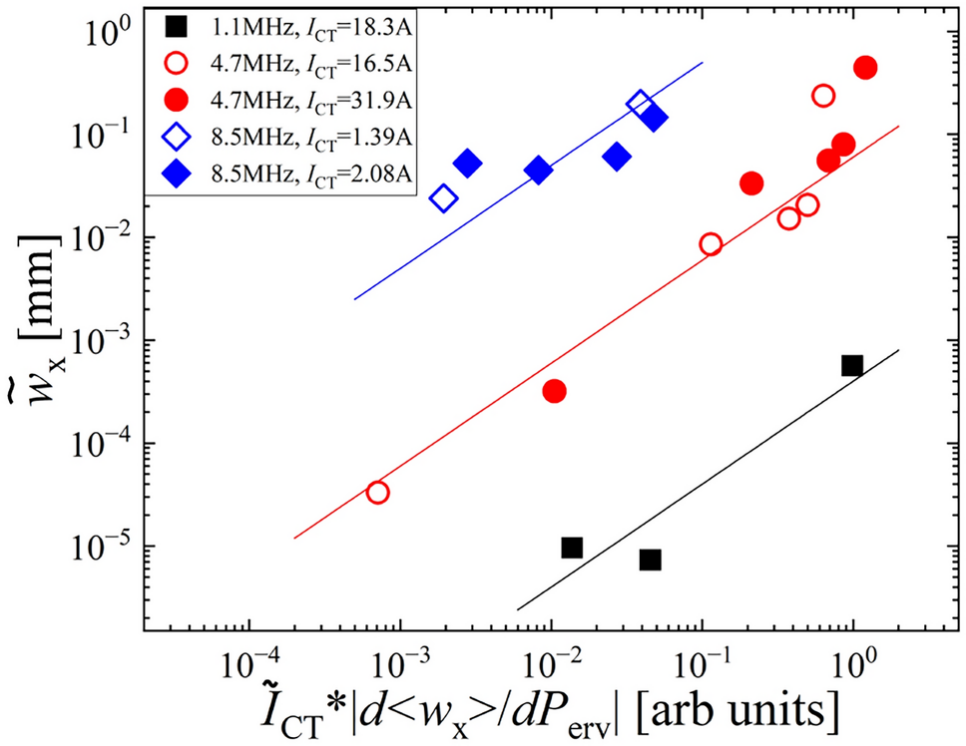
Dependence of the oscillation amplitude of the beamlet width on the gradient of the perveance curve.

In order to investigate the beamlet behavior, a horizontal beamlet profile was analyzed with Gaussian fitting,

1 $$\begin{aligned} J(x)=a \exp \bigg [ -\frac{(x-\mu _{\textrm{x}})^2}{w_{\textrm{x}}^2}\bigg ] +J_{\textrm{c}}, \end{aligned}$$

where *a*, $\mu _{\textrm{x}}$, $w_{\textrm{x}}$ and $J_{\textrm{c}}$ were the peak current density, the position of the beamlet axis, the e-folding half width of the beamlet and the offset, respectively. The time evolution of the parameters obtained by the Gaussian fitting is shown in Fig. 2b-1–b-4. An RF with a frequency of 4.74 MHz was applied to the plasma near the PG and the CT signal is shown in Fig. 2b-1. The oscillations with the perturbation RF frequency were observed in all three parameters, *a*, $w_{\textrm{x}}$, and $\mu _{\textrm{x}}$, which are shown in Fig. 2b-2–b-4. The peak-to-peak amplitude of the beamlet width oscillation was up to $20 \%$ of the averaged beamlet width, which reflects a change in the time-averaged beam divergence. The oscillation amplitude of the beamlet axis position was up to $8 \%$ of the averaged beamlet width, which also directly degraded the beamlet optics.

Regarding the plasma production effect, the total current of the isolated beamlet could not be measured in this experiment because of the limited profile measurement in the vertical direction. However, the out-of-phase relation between the beamlet amplitude oscillation and the beamlet width oscillation indicates that the total current oscillation is small. Another piece of evidence is that no oscillation with the second harmonic frequency was observed. Therefore, the plasma production effect is not considered dominant in this experiment.

In the case of RF positive ion sources, these oscillations were not observed in a core region of the positive ion beamlet, in which the perturbation RF was applied between the PG and the plasma^21^. This is consistent with the fact that the beam divergence angle of the RF positive ion source is almost identical to that of the filament-arc positive ion source. Therefore, the beamlet oscillations observed in this experiment are considered to be a cause of the larger beam divergence angle of the RF negative ion source. The different responses of the sheath boundary to external perturbations indicate a different sheath formation mechanism between negative and positive ion sheaths, which remains for the future. In order to improve the beamlet divergence, the investigation of the dynamic response characteristics of the negative ion beamlet to the RF perturbation is very important.

The RF amplitude dependence and the RF frequency dependence of the beamlet response were investigated and summarized in Fig. 2c-1,c-2. Three frequencies of 1.1 MHz, 4.7 MHz and 8.5 MHz were plotted, and the responses of the beamlet width increased almost linearly with the antenna current (The lines in Fig. 2c-1,c-2 show the linear relation, that is, $\tilde{w}_{\textrm{x}} \propto \tilde{I}_{\textrm{CT}}$ or $\tilde{\mu }_\textrm{x} \propto \tilde{I}_{\textrm{CT}}$, where $\tilde{w}_{\textrm{x}}$, $\tilde{I}_{\textrm{CT}}$ and $\tilde{\mu }_{\textrm{x}}$ are oscillation amplitudes of the beamlet width in the x-direction, RF antenna current and position of the beamlet axis, respectively.). The trend of the beamlet axis position looks similar. The RF amplitude dependencies suggest that the meniscus responds linearly to the perturbation RF electric field because the RF electric field induced by the antenna is proportional to the RF current flowing through the antenna. It is considered that the RF electric field was superposed onto the sheath electric field, and the response of the meniscus to an external perturbation with a small amplitude might be linear. It should be emphasised that the lower frequency might give a solution to mitigate the beamlet oscillation. In a lower frequency range from 1.1 MHz to 4.7 MHz, the oscillation amplitude of the beamlet seems to be proportional to the frequency (Faraday’s law of induction). Therefore, further mitigation might be possible with lower frequency. In a high frequency regime from 4.7 MHz to 8.5 MHz, the frequency dependence becomes stronger. The different frequency dependence would be considered by the change of coupling between the antenna and the plasma, however, further investigation is necessary.

### Perveance dependence

Here, we discuss the perveance dependence of the beamlet dynamics based on the experimental observations, which are shown in Fig. 2c-1,c-2, in which ${\tilde{w}}_{\textrm{x}} \propto {\tilde{E}}_{\textrm{RF}}$. We assume that the beamlet dynamics are determined by the response of the meniscus. In the case of conventional perveance dependence, the beamlet width is determined by the balance between the penetration of the electric field from the beam extraction region to the plasma and the Debye shielding effect of the ion-source plasma. When an external perturbation is applied and the meniscus responds linearly, the change in beamlet width should depend on the gradient of the perveance curve,

2 $$\begin{aligned} {\tilde{w}}_{\textrm{x}} \propto {\tilde{E}}_{\textrm{RF}} \bigg ( \frac{\partial <w_{\textrm{x}}>}{\partial P_{\textrm{erv}}} \bigg ), \end{aligned}$$

where ${\tilde{E}}_{\textrm{RF}}$ and $P_{\textrm{erv}}$ are the perturbation RF electric field amplitude and the perveance ($P_{\textrm{erv}}=I_\textrm{beam}/V_{\textrm{extract}}^{1.5}$, where $I_{\textrm{beam}}$ is the beam current and $V_{\textrm{extract}}$ is the extraction voltage), respectively. The idea of Eq. 2 is sketched in Fig. 3a. It should be noted that the ${\tilde{E}}_{\textrm{RF}}$ corresponds to the oscillation of the meniscus and is not $\tilde{P}_{\textrm{erv}}= {{\tilde{I}}_{\textrm{beam}}}/{V_{\textrm{extract}}^{1.5}}$.

Figure 3b shows the perveance dependence of the beamlet width without the RF perturbation. One can see the perveance matching and over-focus regions due to the weak Debye shielding effect. A similar dependence can be seen in the oscillation amplitude of the beamlet width when the RF perturbation is applied, which is shown in Fig. 3c. In order to investigate the validity of the hypothesis shown in Eq. 2, the amplitude of the beamlet width oscillation is compared with the gradient of the perveance curve ${\partial <w_{\textrm{x}}>}/{\partial P_{\textrm{erv}}}$, which is shown in Fig. 4. The lines in Fig. 4 indicate the linear relation shown in Eq. 2. It was found that the experimental data in low-frequency cases ($f_{\textrm{RF}} = 1.1$ MHz and 4.7 MHz) agree with Eq. 2, although the dependence looks weaker in high-frequency cases ($f_{\textrm{RF}} = 8.5$ MHz). This suggests that the oscillation of the beamlet width can be understood by the direct response of the sheath boundary to the perturbation RF electric field. The RF frequency dependence is also seen, and the lower RF frequency provides the smaller amplitude of the beamlet width oscillation. From the viewpoint of improving beam optics, it should be noted that the oscillation of the beamlet width can be suppressed at the perveance matched condition (${\partial <w_\textrm{x}>}/{\partial P_{\textrm{erv}}} \sim 0$) and lower RF frequency for source plasma production.

In conclusion, the direct responses of the negative ion beamlet divergence and the axis deflection to the RF field were observed to degrade the beamlet optics. It was also established that the response of the beamlet width could be suppressed by the beam operation with the perveance matching condition and with lower RF frequency. It should be noted that the response of the beamlet width to the RF field showed different properties from the response to bias voltage or extraction voltage because the beam current density change was small^22,23^. Regarding the oscillation of the beamlet axis, there seem to be two possibilities. One is the asymmetry of the static meniscus structure caused by the magnetic field effects on the negative ion dynamics in the beam extraction region, which is also consistent with beam transport simulations showing asymmetric meniscus surfaces and/or non-uniform negative ion current density at the meniscus for the same configuration as the present experiment^18,24^ and for different configurations^25,26^. The other is the RF field interaction with the background magnetic field in y-direction ($B_{\textrm{0y}} \sim 0.01$ T). Although the ions cannot move in the *x*-direction responding to RF field because the ion cyclotron frequency ($f_{\textrm{ci}} \sim 1.5 \times 10^{5}$ Hz) is smaller than the RF frequency, the electrons can move in the *x*-direction via $E \times B$ drift, which could cause the beamlet axis to respond to the RF field. The RF frequency dependence of the beamlet axis oscillation observed in Fig. 2c-2 implies that the asymmetry of the meniscus may dominate it and indicates that the response of the beamlet axis can also be suppressed with lower RF frequency.

## Methods

### NIFS-NBTS

This study was carried out using the negative ion beam test stand at the National Institute for Fusion Science (NIFS-NBTS), in which the full-size negative ion source for the LHD plasma experiment can be operated with full specification of the negative ion source.

### NIFS-RNIS

**Fig. 5:**
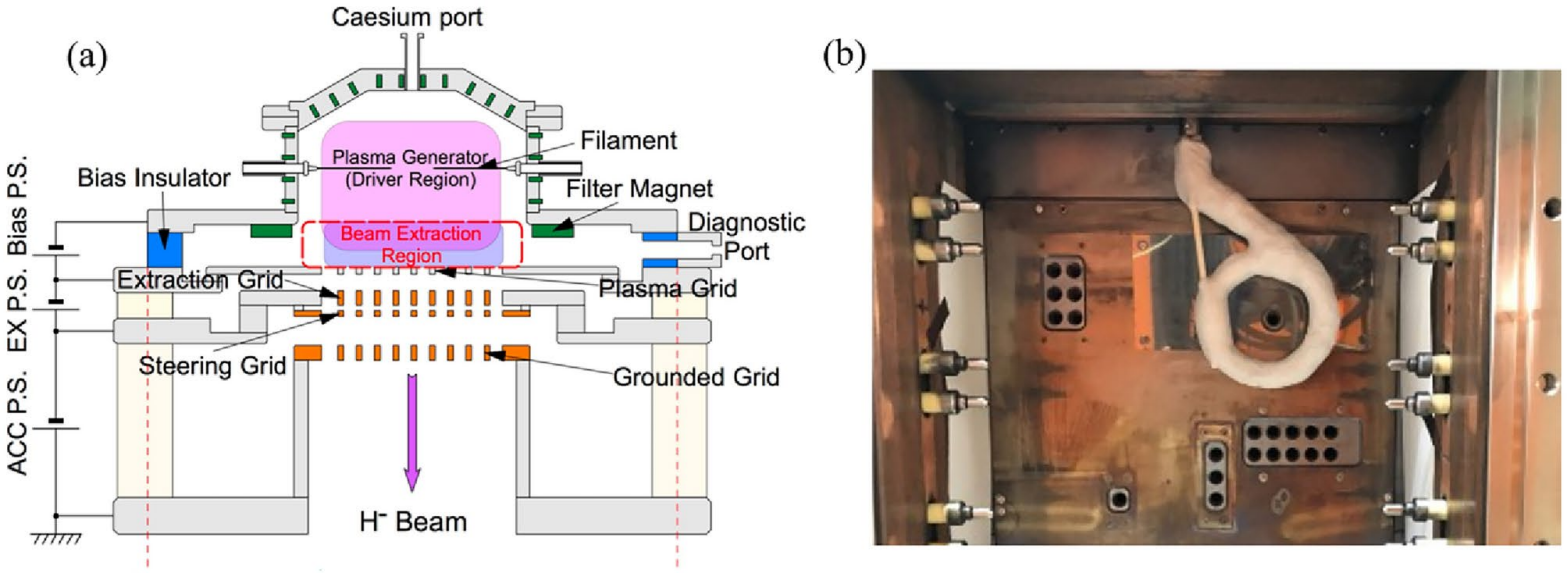
(**a**) Drawing of the horizontal cross-section of RNIS^27^. (**b**) Photograph of the inside of the RNIS with installed RF antenna and PG mask. The beamlet produced through the isolated PG aperture at the center of the RF antenna was measured with FBM in this experiment.

The NIFS-RNIS is for research and development of negative ion sources (see Fig. 5a, which is operated at NIFS-NBTS. The size of NIFS-RNIS used in this study was one third of the negative ion source for the LHD plasma experiment. The inner volume of the arc chamber is $700 \times 350 \times 220$ mm. The beam accelerator consists of two segments of a four-grid system. The beam extraction area of each segment is approximately $250 \times 250$ mm. The accelerator configuration is almost identical to that of the negative ion source of the LHD, a circular multi-aperture for the plasma grid (PG), an extraction grid (EG) with an electron deflection magnet, a steering grid (SG) and a multi-slot type grounded grid (GG). Negative ions were produced mainly by the process on a caesium-seeded PG surface. In order to monitor a single isolated beamlet, the mask plate was mounted onto the PG.

### RF system

The RF perturbation field was applied with an RF antenna installed in NIFS-RNIS (see Fig. 5b). The distance from the PG was around 5 mm. The direction of the RF electric field was perpendicular to the PG. The RF signal with a power level of mW was produced by a signal generator. The RF signal was amplified with a factor of $10^6$ (a gain of 60 dB) using an RF power amplifier (R&K, CA009251-5959R). The amplifier’s maximum power was 1 kW, and its frequency range was from 9 kHz to 250 MHz. In this study, the maximum RF power of 1 kW and a frequency range of from 1.1 MHz to 8.5 MHz were utilized. A $\pi$-type RF matching circuit was developed and used for impedance matching of RF power to the RF antenna. The RF power to the RF antenna was monitored by a current transformer (CT) with high-time resolution, which was inserted between the antenna and the impedance-matching circuit. The RF electric field generated by the RF antenna was measured before installation into the NIFS-RNIS. The maximum RF electric field was roughly 3 kV/m, with 1 kW of RF power to the antenna and 1MHz of frequency.

### Fast beamlet monitor

The beamlet profile was measured with a fast beamlet monitor (FBM) installed at 0.91 m downstream from the GG. The FBM consisted of an $8 \times 4$ ch array of current monitors in horizontal and vertical directions, respectively. The bias voltage of $+ 70$ V was applied to the collector grid for suppression of the secondary electron current, so the absolute values of the negative ion beam current density could be measured. The time resolution of the FBM diagnostic was 25 MHz; therefore, the beamlet’s responses to the RF perturbation could be monitored.

## Data Availability

The data that support the findings of this study are available from the corresponding author upon request.
